# Outcome of Rigo-Chêneau type brace treatment for adolescent idiopathic scoliosis - using the Scoliosis Research Society brace studies inclusion criteria

**DOI:** 10.1186/1748-7161-8-S1-O44

**Published:** 2013-06-03

**Authors:** T Maruyama, H Yamada, Y Nakao

**Affiliations:** 1 Dept of Orthopaedic Surgery, Saitama Medical Center, Saitama Medical University, Saitama, Japan

## Background

We have been using Rigo-Chêneau type brace for the treatment of idiopathic scoliosis since 2007. Curves other than the upper thoracic main curve were the subjects of the treatment. To analyze the outcomes of the brace treatment, use of the standardized inclusion criteria is essential.

## Aim

To evaluate outcomes of Rigo-Chêneau type brace treatment using the Scoliosis Research Society (SRS) AIS brace studies inclusion criteria, which includes patients with age 10 years or older when the brace is prescribed, Risser 0-II, primary curve magnitude 25°-40°, and no prior treatment.

## Results

A total of 32 patients, 27 females and 5 males, met the SRS inclusion criteria. Average age at the beginning of the treatment was 12.0 years (10 to 15). Risser sign was 0 in13, I in 7, and II in 12 patients. Curve pattern was thoracic (T) in 12, thoracolumbar or lumbar (TL) in 12 and double (D) in 8 patients. Average Cobb angle before treatment was 30.9°. Initial correction rate by the brace was 53.5% on an average (42.4% for T, 77.4% for TL, and 34.8% for D curve). Most patients wore their brace as part-time, at home or at night. The average follow-up period was 19 months. Of 32 patients, 15 reached skeletal maturity during the treatment period. Four of them (27%) progressed more than 6°, and two of them (13%) exceed 45°. Only one patient underwent surgical treatment during the study period.

## Conclusion

Although the number of patients who reached skeletal maturity was relatively small, 73% of their curve could be stabilized by the treatment. Rigo-Chêneau type brace was effective for the treatment of adolescent idiopathic scoliosis.

## References

[B1] Richards BS Bernstein RM D'Amato CR Thompson GH Standardization of criteria for adolescent idiopathic scoliosis brace studies: SRS Committee on Bracing and Nonoperative Management Spine 2005 30 18 2068 2075 discussion 2076-2067 10.1097/01.brs.0000178819.90239.d0 16166897

